# Enhanced autosuccession after wildfire in a transitional sub-Mediterranean forest ecosystem: evidence from the Kras Plateau (Slovenia)

**DOI:** 10.3389/fpls.2026.1772621

**Published:** 2026-03-04

**Authors:** Andraž Čarni, Mateja Breg Valjavec, Lucia Čahojová, Aljaž Jakob

**Affiliations:** 1 Jovan Hadži Institute of Biology, Research Centre of the Slovenian Academy of Sciences and Arts, Ljubljana, Slovenia; 2 School for Viticulture and Enology, University of Nova Gorica, Nova Gorica, Slovenia; 3 Anton Melik Geographical Institute, Research Centre of the Slovenian Academy of Sciences and Arts, Ljubljana, Slovenia; 4 Institute of Botany, Plant Science and Biodiversity Centre, Slovak Academy of Sciences, Bratislava, Slovakia; 5 Department of Biology, Biotechnical Faculty, University of Ljubljana, Ljubljana, Slovenia

**Keywords:** autosuccession, forest, Kras Plateau, recovery, sub-Mediterranean, trait, vegetation, wildfire

## Abstract

**Introduction:**

Wildfires are becoming an increasingly prevalent phenomenon in sub-Mediterranean regions, including areas where the vegetation is not historically adapted to fire. However, post-fire successional dynamics in these regions remain poorly documented.

**Methods:**

Vegetation was monitored annually for three years (2023–2025) following a major wildfire on the Kras Plateau (SW Slovenia) in 2022. Monitoring was conducted in 50 permanent plots assigned to five fire-severity classes, including unburned control plots (class 0) and four burned classes (classes 1, 2a, 2b, and 3). We analyzed species composition, vegetation structure, ecological indicator values, species origin and habitat preference, and plant functional traits using ordination and trait-based approaches.

**Results:**

Post-fire succession followed an initial floristic composition model and an enhanced autosuccessional pathway across all fire-severity classes. The early dominance of ephemeral and ruderal species declined rapidly, while the abundance of perennial grasses, shrubs, and resprouting woody species increased. Functional traits shifted along the C–R axis of Grime’s CSR strategy framework: from ruderal towards competitive and stress-tolerant, and successional trajectories consistently converged towards zonal thermophilous deciduous forest communities. High amounts of precipitation facilitated rapid structural recovery, with shrubland developing within three years

**Conclusion:**

Sub-Mediterranean forest vegetation on the Kras Plateau exhibits high resilience to wildfire, despite limited historical adaptation to fire. Enhanced autosuccession, combined with favorable post-fire moisture conditions, enables rapid recovery and reduces the likelihood of long-term degradation or the establishment of persistent post-fire shrublands.

## 1 Introduction

Wildfires significantly affect the natural environment and human lives, and they are a common phenomenon in the Mediterranean region (Pausas et al., 2008). Although Mediterranean ecosystems are resilient to wildfires and climate change, the increased wildfire intensity may impact ecosystem recovery and cause irreversible changes in the vegetation (Ermitão et al., 2024). The frequency of wildfire events is expected to increase also in adjacent regions (Loidi and Vynokurov, 2024; Caucci et al., 2025). In these regions, vegetation is not adapted to wildfires, and dramatic changes in composition and structure of vegetation can be expected (Bussotti and Pollastrini, 2025), especially in coniferous forests, which often spread sub-spontaneously, and exhibit high vulnerability (Arrogante-Funes et al., 2024).

Wildfire is an evolutionary driving force that has shaped plant diversity, their traits, and adaptive strategies in areas prone to wildfires (Lamont et al., 2019). Mediterranean vegetation is often considered highly resistant to wildfires, because repeated fires have led vegetation to adapt to wildfire regimes (Calvo et al., 2013). Many species regenerate vegetatively (resprouters) after wildfires; resprouting is a trait that facilitate vegetation recovery in areas with frequent and severe wildfires (Clarke et al., 2013; Fernández-García et al., 2021). The high resilience of Mediterranean ecosystems is attributed to the long-lasting pre-historical influence of wildfire and Mediterranean vegetation is often dominated by pyrophytes that have adapted to this type of disturbance over time (Naveh, 1975; Le Houérou, 1987; Lamont et al., 2019). However, such events have not been common in the research region, so vegetation is poorly adapted to them. For instance, only two plant species (*Argyrolobium zanonii, Trifolium campestre*) exhibit heat-stimulated germination (Tavşanoǧlu and Pausas, 2018; Čahojová et al., 2024).

As wildfire is a recent phenomenon in our research area, early successional plants have only limited regeneration capabilities, facilitated by traits such as long-distance seed dispersal or efficient resprouting, which significantly influence the recovery pathway. These species can influence the subsequent successional pathways, they can lead to a distinct pathway or favor late-successional species (Vasques et al., 2023). Besides the species composition of the burned vegetation, the severity of the wildfire also plays a critical role in determining the pathway of the recovery process (Trotta et al., 2024).

Succession is a fundamental concept in ecology because it indicates how species populations, communities, and ecosystems change over time (Poorter et al., 2023). The successional pathway in the Mediterranean ecosystem follows the initial floristic composition model (Egler, 1954) and demonstrates that all major species found in the long-unburned control site are already present at the beginning of post-fire succession. The highest species richness is observed two years after the wildfire event (Capitanio and Carcaillet, 2008). Vasques et al. (2023) confirmed that post-fire conditions tend to reach a state of equilibrium similar to that achieved without wildfire, which is consistent with the initial floristic composition model of succession.

Despite extensive research of post-fire succession in Mediterranean ecosystems, there is a notable lack of studies focusing on early successional dynamics in regions at the northern margin of the Mediterranean bioclimatic zone, where wildfires have historically been rare, and vegetation is poorly adapted to fire. There is currently limited knowledge of how vegetation structure, species composition, and functional traits respond to different wildfire severities during the early phases of succession. This knowledge gap hampers our ability to predict future vegetation dynamics and assess ecosystem resilience under increasing wildfire frequency driven by climate change. The 2022 wildfire provided an opportunity to study post-fire succession in this environment.

The study aimed to examine the initial stages of post-fire vegetation recovery. The objectives were to (1) survey the post-fire vegetation recovery, determine the model and pathway of succession and evaluate the speed of successional changes, (2) asses the turnover of Grime’s Competitor-Stress tolerator-Ruderal strategies (CSR strategies), life forms, species origin and habitat preferences, (3) compare vegetation development on plots with varying wildfire severities, and (4) assess trends in ecological conditions within communities and the subsequent successional pathway. We hypothesized that the succession pathway would differ from that in the true Mediterranean region. We expected that the early post-fire recovery stages would be dominated by opportunistic ruderal species, which would later disappear. These species have high nutrient and light demands, a ruderal strategy, and an effective dispersal syndrome. Later, species characteristic of zonal forests would appear. Rather mild climatic conditions would accelerate the recovery pathway. These objectives were addressed by sampling 50 vegetation plots affected by the 2022 wildfire at one, two, and three years after the event, across different levels of fire severity as determined by post-fire vegetation analysis one year after the wildfire.

## 2 Material and methods

### 2.1 Study area

**Figure 1:**
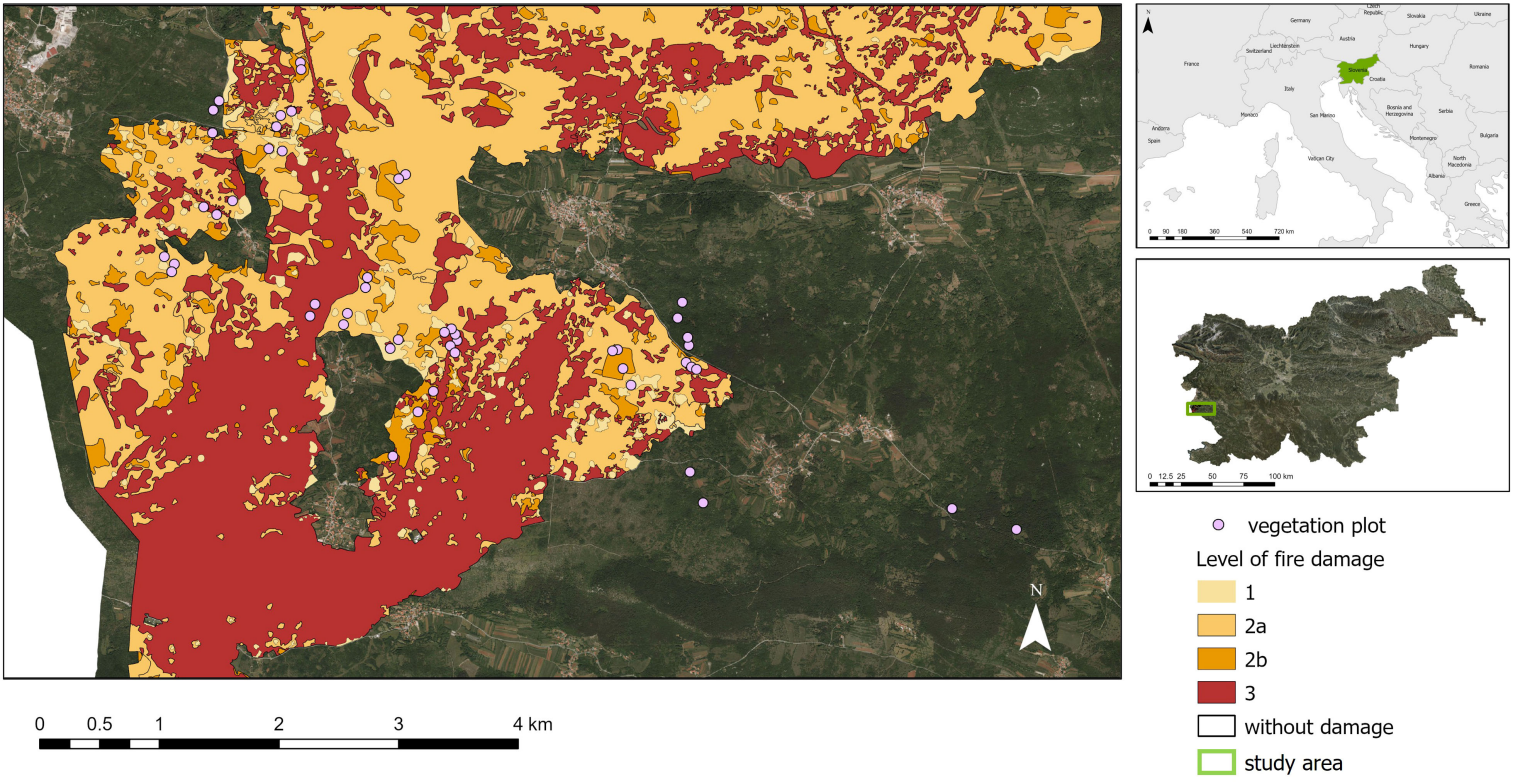
Position of study area in a wider region and extension of wildfire area of the Kras Plateau (south-western Slovenia).

The research was conducted on the Kras Plateau, a limestone karstic plateau located in the northernmost part of the Dinaric Alps above the Bay of Trieste near the Italian-Slovenian border, at an altitude of 200–500 m a.s.l. It consists of karstified Mesozoic limestone, mainly covered by rendzinas and cambisols (Mihevc et al., 2010). Zonal forests are dominated by pubescent oak (*Quercus pubescens*), hop hornbeam (*Ostrya carpinifolia*), and flowering ash (*Fraxinus ornus*) (Jakob et al., 2025). The area has been partially reforested with non-native black pine (*Pinus nigra*), which forms dense communities and spreads sub-spontaneously (Bricca et al., 2025). As the study area is located on the border between the Mediterranean and temperate biomes, the climate is transitional between Mediterranean and continental (sub-Mediterranean), with rainy, cool winters and hot summers (Dinerstein et al., 2017; Barčić et al., 2022). A long and significant dry period here caused the ignition of a major wildfire that broke out in mid-July 2022 and lasted until early August 2022 (Košiček et al., 2022) (**Figure 1**).

#### 2.1.1 Climate data

**Figure 2:**
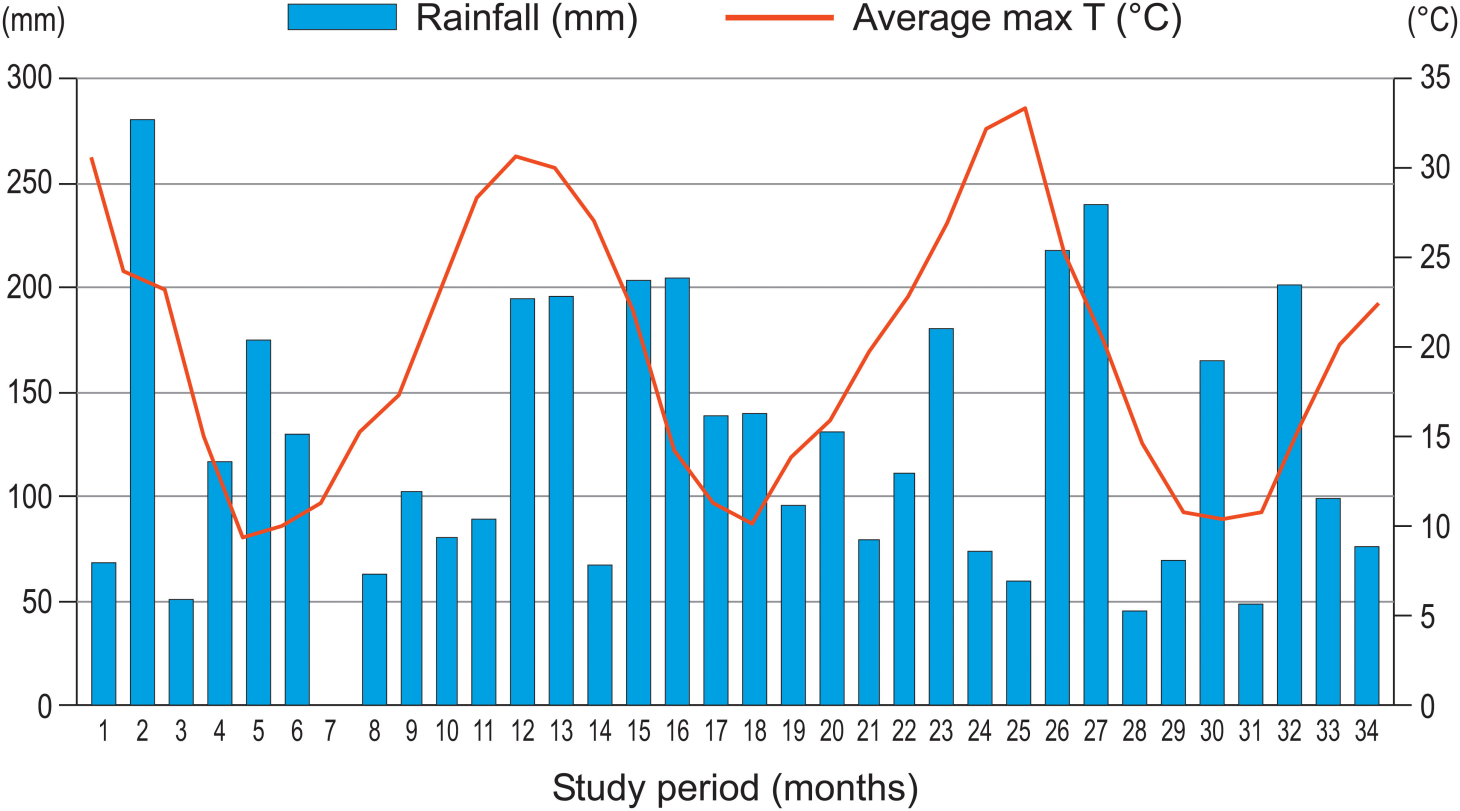
Meteorological conditions after wildfire 2022 related to sampling period 2023/2025. Monthly average maximal temperature and rainfall in 34-month period August 2022/May 2025 at Bilje meteorological station near Nova Gorica. (Source: Slovenian Environmental Agency).

The climate data for the nearby station at Bilje near Nova Gorica for the period August 2022 to May 2025 show marked seasonality: monthly averages of daily maximum temperatures are the highest in summer (peak in August 2024: 33,3 °C), while monthly precipitation totals are more variable, with pronounced peaks mainly in early autumn and also with individual very dry months (e.g., February 2023). The total annual rainfall was 1468.2 mm in year 2023 and 1439.4 mm in year 2024, and average annual temperature was 14.1 °C in 2023 and 14.3 °C in 2024 (**Figure 2**).

### 2.2 Data collection

Permanent plots have been established in this area and basic vegetation sampling was performed in 2023 (Čahojová et al., 2024). Vegetation was resampled in May/June, 2024 and 2025. The plots (10 m × 10 m) are located in areas that were previously dominated by zonal vegetation (*O. carpinifolia-Q. pubescens)*. The plots were selected on the flat parts of the karstic plateau, outside dolines. This minimized the impact of geodiversity and microclimate (Kavgacı et al., 2016). We recorded all vascular plants in the plots by layers and visually estimated the cover of vegetation layers and bare rock in percentage, as well as individual plants in each plot using the 7-degree scale of the Central European method (Braun-Blanquet, 1964).

#### 2.2.1 Estimation of wildfire severity

The severity of the wildfire was estimated by photo interpretation and field observation by the Slovenia Forest Service, and a map of the burned area was prepared (Košiček et al., 2022; Zupan et al., 2023). This map was verified using Sentinel-2 satellite data and the Burn Area Index for Sentinel-2 (BAIS2) (Filipponi, 2018). Burned forests in the area were divided into four severity classes for estimating damage, ranging from 1 to 3. For our study, we added the fifth class: unburned forests (control), category 0, with no fire. Unburned plots were selected in close proximity to the burned ones (**Table 1**).

**Table 1:** Severity class for estimating damage in forests after the 2022 wildfire according to Košiček et al. (2022).

Severity class	Damage
3	Surfaces damaged by a crown or complex fire. This type of fire occurs in the tree crowns, burning the entire above-ground part, including the crowns and trunks. The damage to the forest exceeds 90%.
2a	Surface damaged by fire, transitional between crown and ground fire, with damage greater than 50% and less than or equal to 90%. The fire did not spread to the crowns, but the trunks were damaged. The majority of trees have dried out or are estimated to definitely dry out.
2b	Surface damaged by fire, transitional between crown and ground fire, with damage greater than 10% but less than or equal to 50%. The fire did not spread to the crowns, and the trunks are partially damaged. We estimate that a large proportion of trees affected by the fire will survive.
1	Surfaces damaged by ground fire (damage less than or equal to 10%). Ground vegetation, shrubs, and humus layer are burned. Trees are slightly affected.
0	Unburned.

### 2.3 Data treatment

The vegetation data were stored in the TURBOVEG database (Hennekens and Schaminée, 2001) and transferred to the JUICE 7.1 program for analysis and processing (Tichý, 2002). The JUICE program is specialized software designed for editing and analyzing vegetation data, and was used for the manipulation and analysis of data. It supports various analytical techniques, such as Detrended Correspondence Analysis (DCA), diagnostic species identification, the calculation of bioindicator values, and facilitates the creation of synoptic tables.

#### 2.3.1 Table and diagnostic species

We created the synoptic table by treating each severity class separately (i.e., 0, 1, 2b, 2a, and 3) and calculating the diagnostic species for each year. The diagnostic species were determined by calculating the fidelity of each species to the vegetation plots in each year using the φ-coefficient as a measure of fidelity (Tichý, 2002). We were considering species with a φ value above 0.10 to be diagnostic. Those species whose occurrence in the vegetation plots of a given group was not significant at p < 0.05 (Fisher’s exact test) were excluded from the group of diagnostic species. The table shows the turnover of species in each wildfire severity class. Some of the most common species were then sorted in order of decreasing occurrence below the diagnostic species. We created the synoptic table using the JUICE program (Tichý, 2002).

#### 2.3.2 Ordination

We performed a Detrended Correspondence Analysis (DCA) on the matrix of vegetation plots (Hill and Gauch, 1980). DCA was chosen, as the species response data show unimodal response and the gradient along first axis is more than 2.5 SD long. This reduced the complexity of the data, visualized the relationships between the vegetation plots, and enabled us to identify the gradient within the data (Šmilauer and Lepš, 2014). The cover values of the plant species were converted into percentages and subjected to a logarithmic transformation. This procedure converted the original Central European 7-degree scale into an ordinal scale and assigned adjusted weights to the species cover. This procedure was need as few dominant species might mask the pattern of the rarer species and transformation change the contribution of individual species (van der Maarel, 1979). We present a diagram of the vegetation plots showing the passively plotted cover of the tree, shrub, and herb layer, and bare rock cover on the surface, as well as the unweighted values of the bioindicator values. Calculations were performed using the mass module of the vegan package (Oksanen et al., 2025) in the R environment (R Core Team, 2025).

#### 2.3.3 Indicators of successional pathway

We used several indicators to evaluate changes along the autosuccession pathway. We used the bioindicator values to evaluate the ecological conditions, CSR strategies reflect the adaptation of communities to stress and disturbance, life forms show type of ecosystems (e.g. forest, shrubland, grassland), the origin of species is presented by chorotypes and habitat preference shows the optimal habitat of species.

##### 2.3.3.1 Ecological conditions

Bioindicator values were used for ecological interpretation of the vegetation pattern. The bioindicator values proposed by Pignatti (Pignatti et al., 2005) were used, as they are the most appropriate for our research area. Unweighted mean bioindicator values were calculated for each plot using the JUICE program. The bioindicator values were then passively projected onto the DCA diagram.

##### 2.3.3.2 CSR strategies

We grouped species by strategy type, defined as the role of a plant species within the community, which provides information on the mechanisms underlying species assembly and helps clarify the ecological functioning of the communities. The strategies express the response of species to disturbance and stress conditions (Liu et al., 2025). We calculated the community-weighted mean (CWM) for each vegetation plot using the CANOCO program (ter Braak and Šmilauer, 2012) with binarized species presence, and presented the results in Grime’s CSR triangle (Pierce et al., 2017) as competitors (c), stress tolerants (s), ruderals (r), and their combinations (csr, sr, and cs). We binarized species presences, because dominant species (e.g. *F. ornus*, *Cotinus coggygria*) presented along the whole recovery pathway, would mask changes in strategies. The data were provided by the Biolflor database (Klotz et al., 2002).

##### 2.3.3.3 Life form

The Raunkiær system classifies vascular plants into life forms based on the position of their renewal buds during periods when growth is unfavorable. The system provides information about plant structure, such as phanerophytes, nanophanerophytes, chamaephytes, hemicryptophytes, and therophytes which reflect the type of community along the post-fire recovery pathway (Raunkiaer, 1934). We calculated the percentage of life forms in each vegetation plot according to the Raunkiær system (Midolo et al., 2024). Selected life forms, such as phanerophytes and therophytes are presented as Box-Whiskers diagrams.

##### 2.3.3.4 Origin of species

Chorotypes indicate the geographical origin of the plant species (Fattorini, 2015). In moister, cooler, and more stable climates, species from temperate and boreal regions are expected to appear. In contrast, in burned areas with drier, warmer conditions and pronounced seasonality, more Mediterranean and cosmopolitan species are likely to be found. Cosmopolitan species are more common on man-made and degraded sites. We categorized the plants into chorotypes according to the classification proposed by Pignatti (Pignatti, 1982). We accepted his groups, except we combined steno-Mediterranean and euri-Mediterranean species into a single category: Mediterranean species.

##### 2.3.3.5 Habitat preferences of species

We also calculated the percentage of species according to their habitat preferences (Chytrý et al., 2020). This was estimated using the phytosociological placement of species within the syntaxonomic system, which reflects their ecological conditions, dynamics, and relationships with other species in the plot (Mucina et al., 2016; Troiani et al., 2016). We calculated the percentage of species with the following habitat preferences: species in forests (FAG, PUB, POP, QUE), shrublands (RHA, ROB), grasslands and heaths (MOL, TRI, SED, COR), dry grasslands (FES), and anthropogenic vegetation, as annual (RAP, SIS, CHE, DIF, POL) and perennial (ATR, EPI). We followed the species assignment proposed by Mucina (Mucina et al., 2016) and supplemented it with the species assignment proposed by Poldini (Poldini, 1989). The three-letter code in brackets indicates the assignment of species to phytosociological classes according to Mucina et al. (2016).

#### 2.3.4 Correlation of DCA axes with structural characteristics and ecological conditions

We calculated the Spearman correlation of plot scores on the first two DCA axes with the percentage cover of tree, shrub, and herb layers, as well as the percentage of rock, using the program Statistica (StatSoft, Inc, 2011). Due to the circularity of the bioindicator values (nutrients availability, temperature, moisture, light, and reaction), a modified permutation test was used. We performed a parametric test assuming a normal distribution of the data; a permutation test hypothesizing no relation between the bioindicator values and the scores on the axes; and a modified permutation test accounting for this relation by randomizing the bioindicator values among the species. Only the results of the modified permutation test are presented in the Results section. Spearman correlation for the bioindicator values was calculated using the envfit.iv function in the JUICE program (Zelený and Schaffers, 2012; Zelený, 2018).

#### 2.3.5 Regression of plant traits

We tested the relationships between the environmental gradients represented by the first two DCA axes and the CSR strategies, life forms, species origins, and habitat preferences of species. The first two DCA axes were the independent variables, while CSR strategies, life forms, species origin, and habitat preferences were the dependent variables. We used linear regression, one of the most frequently used techniques (Lord et al., 2025) by the lm function in basic R software (version R 4.4.3) (R Core Team, 2025).

## 3 Results

### 3.1 Table and diagnostic species

The synoptic table (**Table 2**) and the complete analytical table (**Supplementary Table S1**) show that the dominant zonal forest species – *Q. pubescens*, *O. carpinifolia*, and *F. ornus* – are present in the vegetation plots of all wildfire severity classes. We cannot identify any diagnostic species characteristic of individual years in unburned and slightly burned plots (fire intensity class 0 and 1), as there is minimal turnover. The species composition of these vegetation plots remains practically the same, minor changes are result of fluctuations. However, species turnover is clearly evident in burned plots (fire intensity classes 2 and 3).

**Table 2:** Synoptic table of vegetation plots.

Year	2023	2024	2025	2023	2024	2025	2023	2024	2025	2023	2024	2025	2023	2024	2025
Wildfire severity class	0	0	0	1	1	1	2b	2b	2b	2a	2a	2a	3	3	3
Number of plots	11	11	11	2	2	2	15	15	15	12	12	12	10	10	10
*Taraxacum erythrospermum*	27	18	18	50	.	.	80	40	33	42	33	42	50	50	50
*Sonchus asper* ssp. *glaucescens*	.	.	.	100	.	.	93	33	7	67	42	17	60	80	60
*Muscari botryoides*	45	18	36	50	50	50	47	13	13	33	8	8	60	50	20
*Allium sphaerocephalon*	9	.	.	100	.	.	47	7	13	8	.	17	60	20	.
*Crepis setosa *	.	.	.	.	.	.	47	.	.	42	.	.	10	.	.
*Chenopodium album*	.	.	.	.	.	.	20	.	.	8	17	.	20	.	.
*Aegonychon purpurocaeruleum*	.	.	.	.	.	.	20	.	.	.	.	.	.	.	.
*Erigeron canadensis*	.	9	.	.	.	.	.	60	53	.	67	58	.	100	90
*Eupatorium cannabinum*	.	9	.	.	.	.	.	27	7	.	33	25	.	.	.
*Prunus mahaleb*	82	82	82	100	50	100	20	20	60	50	58	67	20	50	80
*Sonchus asper* ssp. *glaucescens*	.	.	.	100	.	.	93	33	7	67	42	17	60	80	60
*Ornithogalum orthophyllum* ssp. *kochii*	9	9	9	.	.	.	27	.	13	42	.	17	40	20	.
*Crepis setosa *	.	.	.	.	.	.	47	.	.	42	.	.	10	.	.
*Medicago prostrata*	.	.	.	.	.	.	7	13	.	25	.	.	40	.	.
*Trifolium incarnatum*	.	.	.	.	.	.	.	.	.	33	8	.	20	20	.
*Allium vineale*	.	.	.	.	.	.	.	.	.	25	.	.	.	.	.
*Erigeron canadensis*	.	9	.	.	.	.	.	60	53	.	67	58	.	100	90
*Medicago lupulina*	.	.	.	.	.	50	13	20	.	8	58	25	20	40	40
*Sonchus oleraceus*	9	9	.	.	.	.	.	7	.	8	58	33	.	.	.
*Rubus ulmifolius*	64	73	64	100	100	100	60	87	93	42	83	100	30	90	100
*Clematis vitalba*	55	55	45	50	50	50	7	53	47	25	83	92	.	70	40
*Lotus corniculatus* ssp. *hirsutus*	.	9	.	.	.	.	13	40	20	17	75	83	40	90	90
*Viola riviniana*	55	45	64	.	50	50	40	40	40	.	25	50	.	30	30
*Torilis arvensis*	.	9	9	.	.	.	13	47	40	.	58	67	.	30	60
*Erigeron annuus*	.	.	.	50	50	.	80	60	40	67	58	33	100	70	40
*Lactuca serriola*	9	.	.	50	50	.	40	7	33	83	67	58	80	90	20
*Allium sphaerocephalon*	9	.	.	100	.	.	47	7	13	8	.	17	60	20	.
*Crocus variegatus*	18	.	9	.	.	.	13	.	.	17	8	8	60	30	.
*Medicago prostrata*	.	.	.	.	.	.	7	13	.	25	.	.	40	.	.
*Lathyrus setifolius*	.	.	.	.	.	.	.	.	.	.	.	.	30	.	.
*Clematis vitalba*	55	55	45	50	50	50	7	53	47	25	83	92	.	70	40
*Senecio vulgaris*	.	.	.	50	.	.	47	13	33	25	17	25	40	70	20
*Cytisus nigricans*	.	.	.	.	.	.	.	33	20	.	17	17	.	20	50
*Crepis foetida*	.	.	.	.	.	.	.	7	.	.	.	.	.	30	.
*Capsella bursa-pastoris*	.	.	.	.	.	.	.	.	.	.	8	.	.	30	.
*Erigeron canadensis*	.	9	.	.	.	.	.	60	53	.	67	58	.	100	90
*Rubus ulmifolius*	64	73	64	100	100	100	60	87	93	42	83	100	30	90	100
*Prunus mahaleb*	82	82	82	100	50	100	20	20	60	50	58	67	20	50	80
*Cornus mas*	55	55	55	100	100	100	47	53	80	50	50	67	.	.	40
*Torilis arvensis*	.	9	9	.	.	.	13	47	40	.	58	67	.	30	60
*Geranium purpureum*	.	.	.	.	.	.	13	33	27	17	25	50	30	60	90
*Crepis pulchra*	.	.	.	.	.	.	13	20	7	.	50	42	.	30	60
*Carduus nutans*	.	.	.	.	.	.	.	7	.	8	17	17	.	40	70
Other species															
*Fraxinus ornus*	100	100	100	100	100	100	100	100	100	100	100	100	100	100	100
*Cotinus coggygria*	100	91	91	100	100	100	100	100	100	100	100	100	100	100	100
*Viola hirta et alba*	100	73	82	100	100	100	87	100	93	100	100	100	100	100	100
*Sesleria autumnalis*	100	100	100	100	100	100	100	100	100	83	75	83	80	90	100
*Brachypodium rupestre*	91	91	91	100	100	100	80	80	93	67	67	83	90	90	90
*Quercus pubescens*	100	100	100	100	100	100	80	80	87	50	58	67	70	70	70
*Fraxinus ornus* burned	.	.	.	50	100	100	100	100	100	100	92	83	100	100	90
*Ostrya carpinifolia*	91	82	91	100	100	100	87	100	100	25	42	58	40	40	50

Layers are merged and species are presented as a percentage presence in individual column. The framed species show fidelity above 0.10, calculated for the specific year within the severity class. The most common species are at the bottom of the table. The full table is in the **Supplementary Material Supplementary Table S1**.

### 3.2 Ordination diagram and recovery pathway

**Figure 3:**
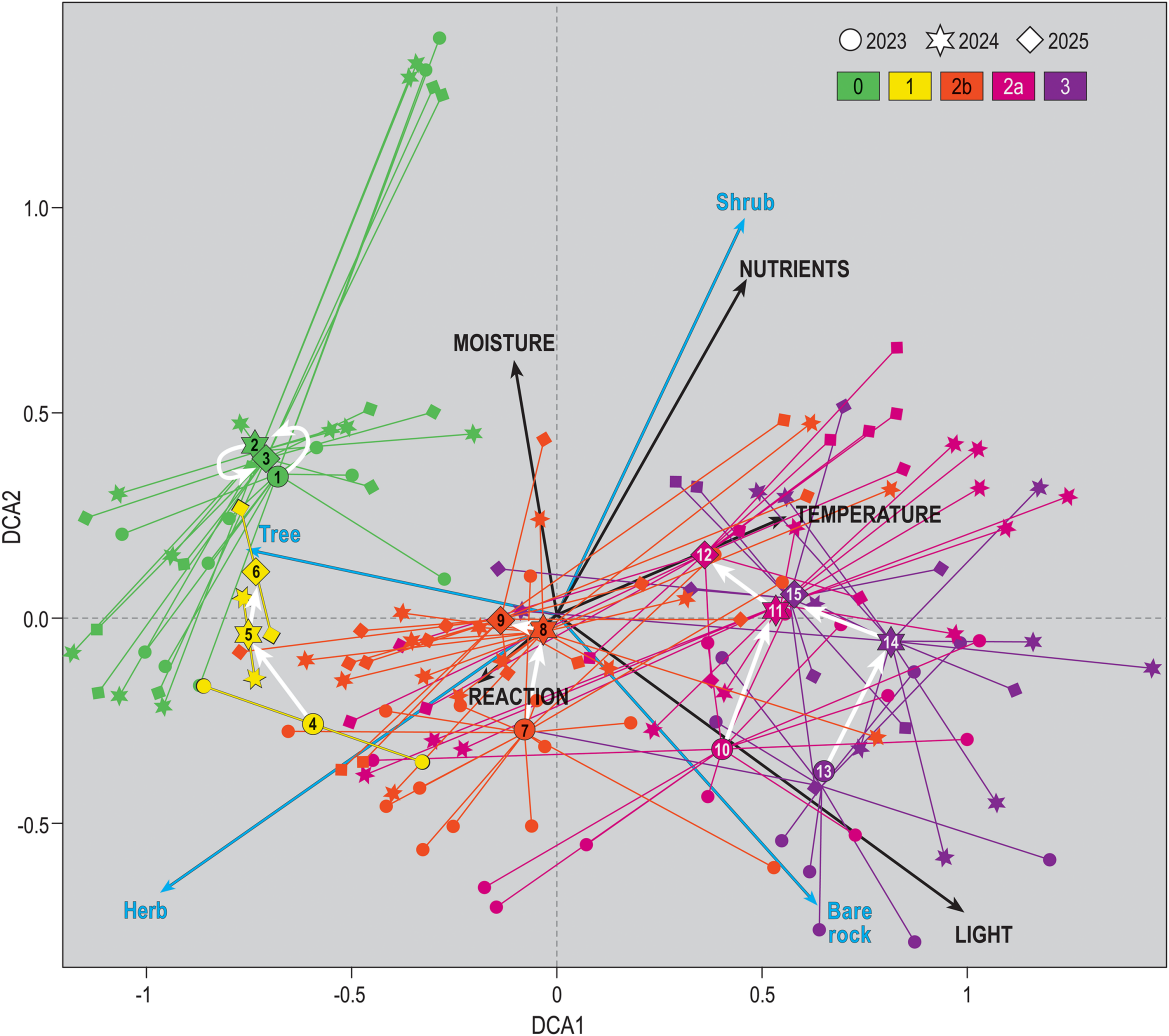
Diagram of Detrended Correspondence Analysis (DCA). Vegetation plots are shown, along with the centroids of plots from a specific year within severity classes. Severity class is indicated by color (severity classes: 0 (green, unburned), 1 (yellow, damage 0–10%), 2b (orange, damage 10–50%), 2a (red, damage 50–90%), 3 (violet, damage 90–100%)), and year by shape (circle/2023, star/2024, diamond/2025). Bioindicator values (light, nutrients, moisture, and reaction), as well as vegetation layers and bare rock, are passively projected onto the diagram plane. Numbers in the centroids correspond to the column numbers in **Table 2**. White arrows indicate successional pathways, except for unburned plots, where these arrows represent fluctuation.

The DCA diagram (**Figure 3**) with passively projected bioindicator values shows that the first axis reflects wildfire severity, while the second axis primarily reflects changes in ecological conditions. The correlation between the first two axes and the structure of vegetation and ecological conditions (**Table 3**) indicates that the first axis represents the wildfire severity gradient. On the left side, unburned plots have a well-developed tree and herb layer and less bare rock on the ground. On the right side, the most severely burned plots are found, with many burned trees, extensive bare rock on surface, and high light availability. Axis 2, in contrast, correlates with vegetation development during the first two years after a wildfire. During this period, the shrub layer develops intensively, there is an increased nutrients and moisture in the stands. The cover of bare rock on the ground decreases, and burned trees and shrubs gradually disappear.

**Table 3:** Spearman correlation of vegetation plot scores on the first two DCA axes with the percentage cover of the tree, shrub, and herb layers, percentage of bare rock, and bioindicator values (nutrients, temperature, moisture, light, and reaction).

DCA1	Correlation	p	DCA 2	Correlation	p
Structural characteristics					
Cover tree layer (%)	-0.282931	***	Cover tree layer (%)	0.135622	
Cover shrub layer (%)	0.14698		Cover shrub layer (%)	0.355035	***
Cover herb layer (%)	-0.548341	***	Cover herb layer (%)	-0.168586	*
Cover bare rock (%)	0.429238	***	Cover bare rock (%)	-0.244748	**
Cover of burned tree layer (%)	0.709067	***	Cover of burned tree layer (%)	-0.352213	***
Cover of burned shrub layer (%)	0.126493		Cover of burned shrub layer (%)	-0.254922	**
Bioindicator values					
light	0.767	***	light	-0.303	
temperature	0.332		temperature	0.081	
moisture	-0.163		moisture	0.489	**
soil reaction	0.392		soil reaction	0	
nutrients	0.22		nutrients	0.58	***

The significance of the p value is indicated as follows: 0 “***” 0.001, “**” 0.01, “*” 0.05, “ “ 1.

#### 3.2.1 CSR strategies

**Figure 4:**
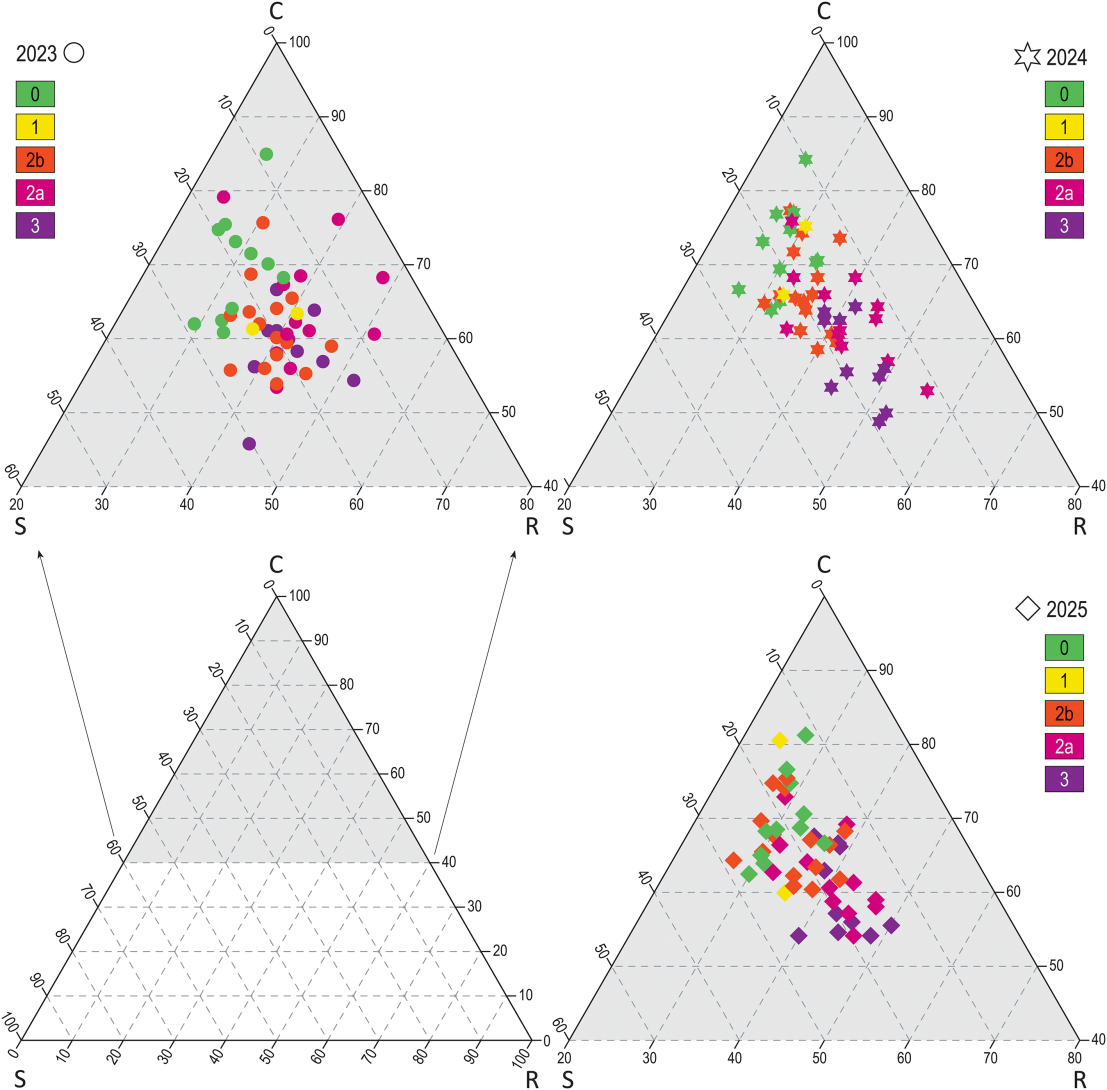
Grime’s CSR triplot. Each year is presented in a different triplot. Severity class is indicated by color (severity classes: 0 (green, unburned), 1 (yellow, damage 0–10%), 2b (orange, damage 10–50%), 2a (red, damage 50–90%), 3 (violet, damage 90–100%)), and year by shape (circle/2023, star/2024, diamond/2025). We can see a shift along the C/R axis among the plots of different severity classes, the proportion of ruderals significantly diminish over years.

Grime’s CSR triplot and regression (**Figure 4**, **Table 4**) show changes in strategies. At the beginning of succession, there is a slight shift from ruderal to competitor species. During the next stage, there is a dramatic decline in ruderals and an increase in competitors and to a lesser extend also stress-tolerant species.

**Table 4:** Linear regression, with the independent variables being the scores of the vegetation plots on the first two DCA axes and the dependent variables being the plant traits (life forms, strategies, chorotypes, and habitat preferences).

	DCA1				DCA2			
Indicator	t-values	AdjR2	p	Significance	t-values	AdjR2	p	Significance
CSR strategies								
competitor	-8.119	0.3035	1.69E-13	***	5.531	0.1657	1.40E-07	***
stress-tolerator	-4.253	0.1029	3.72E-05	***	-4.166	0.09892	5.24E-05	***
ruderal	16.01	0.6315	2.00E-16	***	-2.841	0.04532	5.13E-03	**
Life forms								
chamaephytes	-0.046	-0.00674	0.964		-5.595	0.169	1.04E-07	***
geophytes	-8.307	0.2065	3.12E-09	***	-1589	0.01012	1.14E-01	
hemicryptophytes	1.244	0.003656	0.216		-6.558	0.2199	8.54E-10	***
nano-phanerophytes	-4.312	0.1056	2.94E-05	***	6.869	0.2366	1.67E-10	***
phaneophytes	-6.271	0.2046	3.74E+09	***	8.545	0.3258	1.46E-14	***
therophytes	15.431	0.6141	2.20E-16	***	-1.598	0.01033	1.12E-01	
Chorotypes								
Mediterranean	1.543	0.00919	0.125		1.824	0.01538	7.02E-02	
Mediterranean montane	-4.457	0.1124	1.63E-05	***	-1.922	0.01776	5.65E-02	
Eurasian species	-6.447	0.214	1.52E-09	***	0.17	-0.00656	8.66E-01	
SE mountains	1.895	0.01709	0.06		-1.546	0.009252	1.24E-01	
boreal	-3294	0.06203	0.00123	**	0.042	-0.00675	9.66E-01	
cosmopolitic	12.739	0.5198	2.00E-16	***	0.8	-0.00242	4.25E-01	
Chorotypes								
forest species	-12.33	0.5036	2.00E-16	***	5.767	0.1779	4.55E-08	***
shrub species	-2.428	0.03182	0.0164	*	3.682	0.07771	3.24E-04	***
mesic grassland and fringe species	-2.059	0.02127	0.0413	*	-6.002	0.1903	1.44E-08	***
dry grassland species	3.414	0.06673	0.000826	***	-7.426	0.2665	8.21E-12	***
annual weed species	13.017	0.5306	2.00E-16	***	-1.562	0.009573	1.20E-01	
perenial weed species	12.237	0.4996	2.00E-16	***	1.037	0.000508	3.01E-01	
various Mediterranean habitats	-3.413	0.06669	0.00083	***	4.581	0.1183	7.50E-07	***

The significance of the p value is indicated by the following intervals: 0 “***” 0.001 “**” 0.01 “*” 0.05 “ “ 1.

#### 3.2.2 Life forms

**Figure 5:**
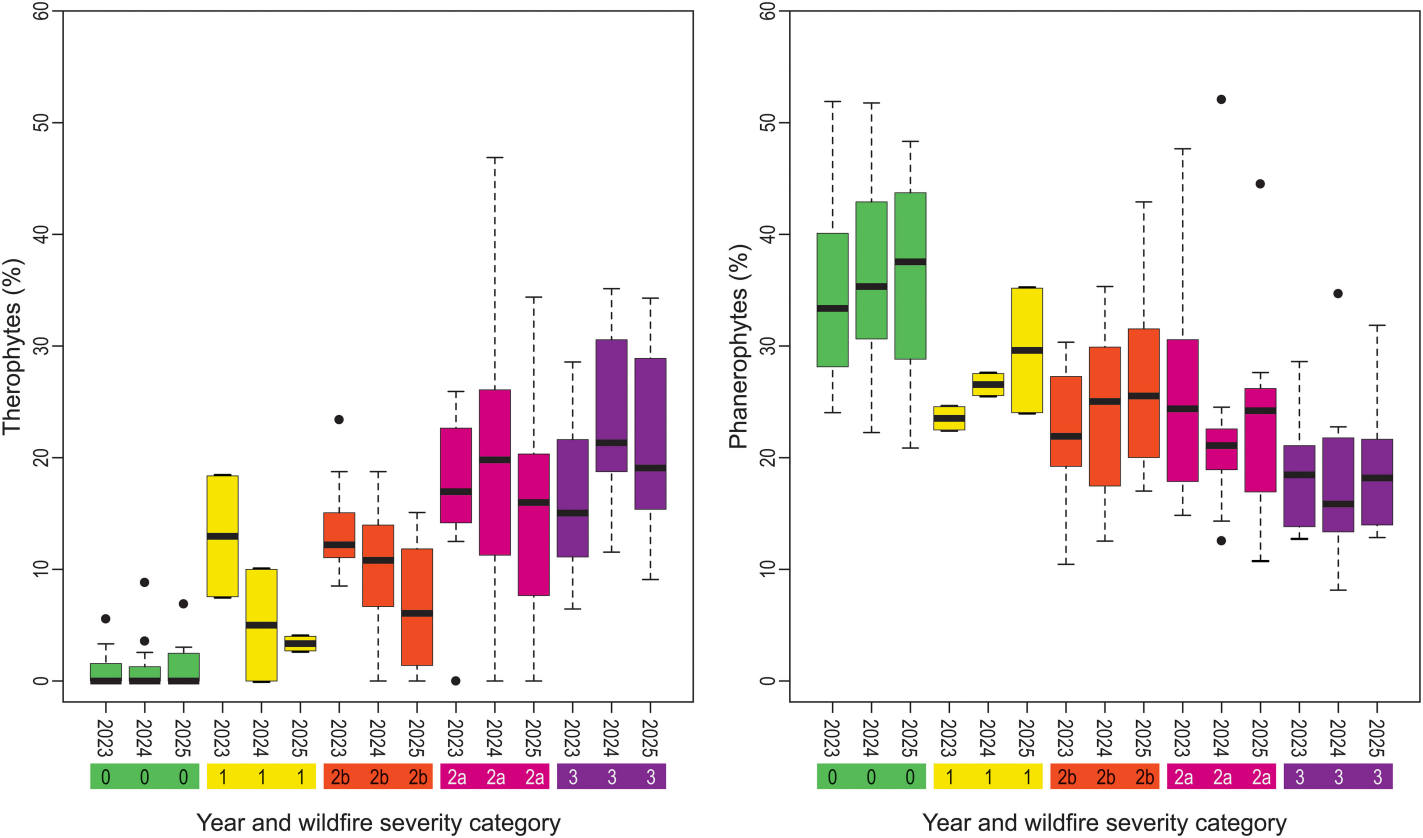
Box-Whiskers diagram showing the proportion of therophytes and phanerophytes in specific years. Severity class is indicated by color (severity classes: 0 (green, unburned), 1 (yellow, damage 0–10%), 2b (orange, damage 10–50%), 2a (red, damage 50–90%), 3 (violet, damage 90–100%)). Therophytes appear rarely in zonal vegetation (green color), but they are present at the beginning of the successional pathway. The inverse is situation with phanerophytes, which are more prevalent in the zonal vegetation.

Raunkier’s life forms (**Figure 5**, **Table 4**) show that woody plant species (nanophanerophytes and phanerophytes) increase during the first two stages. Chamaepyhtes and hemicryptophtes decrease at the beginning and remain relatively stable afterwards. Geophytes and therophytes remain stable initially, but therophytes decrease dramatically and geophytes increase subsequently. As can be seen in **Figure 5** therophytes remain unchanged in the unburned control, decrease continuously in the partially burned area (fire severity class 1 and 2b) and but increase initially before decreasing in the next stage in the severely burned plots (fire intensity class 2a and 3). The similar pattern is seen for phanerophytes in the severely burned plots: they slightly decrease at the beginning but increase afterwards.

#### 3.2.3 Origin of species

The origin of species (chorotypes/chorological spectrum, **Table 4**) does not change at the beginning of succession. It seems that these stands are dominated by opportunistic cosmopolitan species. During the next stage, the proportion of cosmopolitan species decreases, while Eurasian, Mediterranean-montane, and boreal species increase.

#### 3.2.4 Habitat preferences of species

The habitat preferences of species (**Table 4**) show that during the first stage, the proportion of forest and shrubland species increases, as do species from various Mediterranean communities. Species from mesic grasslands and fringes, as well as species from dry grasslands, decrease. The number of annual ruderal and weed species and perennial ruderal species remains practically the same during the first stage. During the next stage, their proportions, as well as proportion of dry grassland species, dramatically decrease. The proportions of forest, shrub, mesic grassland and fringe species, as well as species from various Mediterranean habitats, increase during this stage.

## 4 Discussion

In burned areas, the shrub layer, composed of resprouters (e.g., *C. coggygria*, *F. ornus*) and opportunistic ephemeral species that benefit from the large amount of nutrients released by wildfire, develops first. After two years, when the shrub layer is established and the nutrients are depleted, the tree and herb layers begin to develop. There is less bare rock on the ground, and the communities become denser and more mesic. The successional pathways in plots with different wildfire severities are parallel: after the initial development of ephemeral, nitrophilous vegetation, the vegetation develops toward the potential zonal vegetation. We confirmed most of our hypotheses, except for the model and pathway of succession – it is the same as in the Mediterranean region: initial floristic composition and enhanced autosuccession.

Droughts, heatwaves, and wildfires have been assumed to be risks only in Southern Europe and its Mediterranean, but new analyses and more frequent wildfires events have revealed that climate change affects the intensity, duration, and impact of these events also in the Central Europe (Dolák et al., 2025; Skrobala et al., 2025). The Kras Plateau is located on the border between the Mediterranean and temperate biome, where wildfires have not been common in the past. They are becoming more frequent due to climate change and agricultural land abandonment (Zorn et al., 2024; Tonet, 2025). The 2022 wildfire provided a unique opportunity to define post-fire succession model in karstic environment and develop new knowledge on how vegetation structure, species composition, and functional traits respond to different fire severities during the early phases of succession.

### 4.1 Succession model and pathway

In most cases, post-fire succession in the Mediterranean follows the initial floristic composition model (Egler, 1954). Our analyses show that resprouters largely survived the wildfire and that the understory vegetation mostly recovered within three years, even in the cases of the most severe wildfire (crown fire). The early appearance of nitrophilous ephemeral species, due to increased nutrient and light availability, could indicate species turnover and support the rely floristics model. However, there has been a rapid expansion of clonal graminoids (*Brachypodium rupestre*, and *Sesleria autumnalis*), forming a dense carpet. These species can resprout and possess a dense leaf sheaths for protection and rapid recovery after fire (De Luis et al., 2004; Pilon et al., 2021).

In the years following the wildfire, the landscape is characterized by resprouters such as *C. coggygria* and *F. ornus*, seeders like *Fumana procumbens* (which is present in the seed bank and is fire tolerant), *Lathyrus setifolius* (which is present in the seed bank), geophytes like *Crocus variegatus* (which survive as corms underground), opportunistic ephemeral species such as *Sonchus asper* and *Erigeron annuus* (with good spreading ability), and other species that survived the wildfire (Čahojová et al., 2024). Ash deposited on the ground increases nutrient availability, supporting the growth of opportunistic ephemeral, nutrient-demanding species (Kavgacı et al., 2010; Agbeshie et al., 2022).

The presence of shrubs and trees is directly related to the vegetation that existed prior to the fire, due to post-fire regeneration through resprouting or the establishment of seedlings from a seed bank or from seeds brought by the wind from neighboring forests. Dominant tree species, specially *F. ornus* possess very vivid resprouting ability, but there is no evidence of epicormic resprouting that would enable forests to recover rapidly after wildfire (Pausas and Keeley, 2017; Shaw, 2023). The only problematic tree species is pine (*P. nigra*), which lacks both the ability to resprout and emerging from a seed bank. These stands are highly flammable and should be converted into thermophilous deciduous forests in the future (Diaci et al., 2019; Shaw, 2023; Kavgacı and Keleş, 2025).

The pathway can be considered as an example of autosuccession, in which all species are present in the plot, yet distinct communities (or vegetation types) can still be identified along it. Plant communities change primarily through shifts in species abundance rather than species composition (Hanes, 1971; Kavgacı et al., 2017).

This process cannot be described as direct or cyclic succession (Whittaker and Levin, 1977). The facilitation model (Connel and Slatyer, 1977) is also inapplicable, since short-lived, ephemeral opportunistic species do not facilitate further vegetation development. Furthermore, it is not possible to distinguish different mechanisms along this complex pathway, which comprises many species (Pickett et al., 1987). The most appropriate term is “enhanced autosuccession” encompassing the appearance of early-stage nitrophilous ephemeral species, followed by the development of potential natural vegetation, without further intermediate stages (Buhk et al., 2006).

#### 4.1.1 CSR strategies

CSR strategies are a useful tool for understanding the plant adaptation and the ecological functioning of communities (Liu et al., 2025). During succession, the ruderal CSR strategy is the most dynamic along the pathway, it decreases dramatically during the second stage, with communities shifting towards the CS strategy characteristic of zonal forest vegetation (Stupar and Čarni, 2017; Ricci et al., 2024).

#### 4.1.2 Life forms

According to the life forms classification by Raunkiaer, this is not typical Mediterranean forest vegetation, as the proportion of phanerophytes (trees) and chamaephytes (dwarf shrubs) should increase during recovery in typical Mediterranean vegetation (Carrari et al., 2022). In our case, however, the proportion of phanerophytes decrease slightly in severely burned plots at the first stage. This could be due to the gradual death of damaged trees during the wildfire, difficulties in phanerophyte germination on open sites covered in destroyed soil, or changes in intraspecific relations within communities. But the proportion of phanerophytes generally increases along the pathway (Vilaplana et al., 2024; Calderisi et al., 2025). Chamaephytes decrease and they are only sporadically present in zonal forests in the region (Poldini, 1989). Therophytes increase during the initial stage, but then decrease in the following stages in all other plots, except in plots subject to the severe wildfire (crown fire), where the decrease is delayed. This massive colonization of sites by therophytes is caused by reduced competition, as well as high light and nutrient availability resulting from rapid mineralization during the wildfire, which decreases in subsequent stages (Kavgacı et al., 2010; Ricci et al., 2024).

#### 4.1.3 Origin of species

In the initial stages, there are no changes in chorotypes. The stands are dominated by cosmopolitan species that have an effective dispersal, are widely distributed, and have a wide ecological niche (Pignatti, 1982). During the next stage, there is a significant decline in cosmopolitan species, which are replaced by species characteristic of zonal forests, such as Eurasian and Mediterranean-montane species. These species grow in more humid and shaded sites, are more specialized, and possess a biogeographical signal (Jakob et al., 2025; Koljanin et al., 2025).

#### 4.1.4 Habitat preferences of species

At the beginning of the succession process, ephemeral, nitrophilous annual species appear, which are classified as annual weeds and ruderal species of the class *Stellarietea mediae*. The following year, they are joined by perennial ruderal species of the class *Artemisietea*. At this point, succession towards forest intensifies, firstly with the shrub species of the *Rhamno-Prunetea* and finally with the forest species, mainly from the class *Quercetea pubescentis*. As sub-Mediterranean, thermophilous deciduous forests do not have a closed canopy, species of dry grasslands (*Festuco-Brometea*), forest fringes (*Trifolio-Geranietea*), and others can be found there. The pathway can be summarized as follows: annual ruderal and weed species, perennial ruderal species, shrubland, and forest species (Čarni, 1994, 1998, 2005; Mucina et al., 2016; Čarni et al., 2021).

### 4.2 The influence of environmental factors on succession pathway

In Mediterranean ecosystems, early post-fire succession is tightly constrained by water availability because summers are hot and dry; consequently, the timing and amount of post-fire rainfall strongly control germination, establishment, and survival of juvenile plants, while drought immediately after fire can suppress recruitment of seed-regenerating species and shift the competitive balance toward deep-rooted shrubs and resprouters, potentially slowing or redirecting the successional trajectory (including reduced tree recovery under combined fire damage and drought stress) (Trabaud, 1994).

Environmental conditions can facilitate or hinder the course of natural succession after a wildfire in the Mediterranean climate. The Mediterranean environment is characterized by hot, dry summers, so the availability of moisture after a wildfire greatly affects the survival of young plants. Drought periods immediately after a wildfire can reduce the germination and survival of seed-regenerating species, giving a relative advantage to shrubs with deeper roots and stored reserves. Research shows that post-fire drought combined with wildfire damage can cause high mortality even in species that can otherwise withstand a single wildfire (Capitanio and Carcaillet, 2008; Roche et al., 2024). Stress due to drought can also weaken the recovery capacity of trees (e.g., older oaks may fail to sprout), which inhibits their dominance and allows pioneer species to reign longer. Predictions indicate that under climate change conditions (more frequent droughts), oaks could suffer a greater decline than pines or shrubs, leading to a change in species composition—i.e., more persistent coniferous or shrub phases instead of a transition to a climax forests (Vasques et al., 2023).

In contrast to many Mediterranean case studies described by Carrari et al. (2022), where early succession often remains limited to dwarf shrub or sparse shrubland stages, the Kras Plateau experienced relatively favorable precipitation conditions following the 2022 wildfire, with high annual rainfall totals in both 2023 and 2024 and pronounced autumn rainfall peaks. These conditions likely mitigated post-fire water stress and facilitated the rapid transition from ephemeral nitrophilous species to shrub-dominated communities, as observed in our vegetation data. Similar to the patterns reported by Carrari et al. (2022), early post-fire succession on the Kras was characterized by strong resprouting responses and rapid structural development; however, the higher moisture availability at this northern, sub-Mediterranean margin appears to have accelerated the process. As a result, shrubland developed within three years after the wildfire, indicating an enhanced autosuccessional pathway rather than prolonged stagnation at early successional stages. This comparison highlights that, even in regions where vegetation is not fully fire-adapted, sufficient post-fire precipitation can substantially increase ecosystem resilience and speed of recovery, reducing the risk of long-term degradation or irreversible vegetation shifts.

## 5 Conclusion

We have demonstrated the recovery pathway of sub-Mediterranean thermophilus forests and show some similarities and differences to pathways taking place in a strict Mediterranean region. As the area is situated on the border of the Mediterranean region, the macroclimatic conditions are less severe. This is also reflected in the speed of the recovery pathway: in the third year, we can find already shrubland (structurally comparable to maquis), whereas in the Mediterranean region, only garrigue (dwarf shrubs) can be found (Kavgacı et al., 2010).

As there are a limited number of monitoring sites for post-fire recovery on the margins of the Mediterranean region, where wildfires have become a complex problem, it is important to follow the recovery pathway. Knowledge plays a crucial role in the implementation of wildfire management in these regions.

## Data Availability

The original contributions presented in the study are included in the article/**Supplementary Material**, further inquiries can be directed to the corresponding author.
